# Staff motivation and welfare in Adventist health facilities in Malawi: a qualitative study

**DOI:** 10.1186/s12913-014-0486-4

**Published:** 2014-11-01

**Authors:** Fyson Kasenga, Anna-Karin Hurtig

**Affiliations:** Malawi Union of the Seventh Day Adventist Church, P.O. Box 951, Blantyre, Malawi; Department of Public Health and Clinical Medicine, Umeå University, Umeå International School of Public Health, Epidemiology and Public Health Sciences, SE-901 85 Umeå, Sweden

**Keywords:** Staff motivation, Adventist health facility, Staff development, Malawi

## Abstract

**Background:**

To explore factors that motivate members of staff at Adventist health facilities in Malawi to maximize their potential for work and improve their welfare. This was a qualitative study that utilized group discussions and in-depth interviews with health care staff members.

**Methods:**

Four group discussions with health care workers and support staff were conducted.

**Results:**

Both motivating and demotivating factors were found. The motivating factors were spiritual nourishment of the institutions and working conditions with long term benefits for individuals and their families. The demotivating factors were unfair treatment without respect to staff by management. Specific areas like working condition, housing, allowances, training, communication, and personal support were highlighted as some of the factors that poorly motivated staff to stay at the health facility Further, issues related to the loose of purpose, where Christian values were seen to be deteriorating were observed to be undermining mission of the institutions.

**Conclusions:**

Staff motivation is vital in any working condition in as far as good performance at the work environment is concerned. Poor working conditions have not been exceptions among the Adventist health institutions. Based on these findings, the study recommended that conditions of services for the Adventist health facilities need to be revised and implemented accordingly; training of staff for further facility development to be intensified, communication between management and health care staff through scheduled routine meetings need to be strengthened. Spiritual nourishment through staff interaction with church officials and pastors should always be considered. A further study is needed to look at the community perceptions towards the services offered in the Adventist health facilities.

## Background

In many poor resource settings, health facilities face multiple challenges in order to provide quality care. Resources are of different nature ranging from economic, infrastructure, consumables as well as humans among others. Human capital is one of the most important resources an institution can have. From the time immemorial until the turn of the century, organizations of different capacities have been investing in human resources as one way of sustaining the existence of their operations. In the studies done before, it was found that training of the personnel in any given organization has a long-term impact [1]. The investment of human resources is done in different ways; short term or long-term trainings in whatever form has proved to be expensive but rewarding. Even though different forms of trainings are offered to members of staff of different cadres in an organization, the actual full translation of knowledge and skills into practice remains questionable in certain circumstances. This is dependent on the individuals who participate in such trainings as the common saying goes that “you can take a camel to the river but you can never force it to drink water”.

Motivation has been defined as the driving force by which humans achieve their goals. Motivation is said to be intrinsic or extrinsic [2]. According to various theories, motivation may be rooted in a basic need to minimize physical pain and maximize pleasure in the form of success in whatever kind. Motivation may include specific needs such as eating and resting, or a desired object, goal, state of being, ideal, or it may be attributed to less-apparent reasons such as altruism, selfishness, morality, or avoiding mortality. As institutions have missions and visions, individuals too do have purpose for their existence and work or look towards self actualization. In this case we may deduce that factors that trigger motivation may be abstract or concrete. Despite rural health services being situated and integrated within communities in which people work and live, the complex interaction of the social environment on health workers’ motivation and performance in low and middle income countries has been neglected in research. Razee et al., [3], identified the key social factors impacting on health workers’ motivation and performance to be the local community context, gender roles and family related issues, safety and security, health beliefs and attitudes of patients and community members. They further identified the importance of strong supportive communities and working environment on health worker motivation. Motivation is influenced by both financial and non-financial incentives. The main motivating factors for health workers were appreciation by managers, colleagues and the community, a stable job and income and training. The main discouraging factors were related to low salaries and difficult working conditions.

Motivation goes along side with human welfare and these two concepts move hand in hand and at times they are inseparable. This is because one who is positively motivated tends to work hard towards achieving his/her goals and consequently enjoys what is known as human/staff welfare. Employee welfare is defined as “efforts to make life worth living for workmen”. These efforts have their origin either in some statute formed by the state or in some local custom or in collective agreement or in the employer’s own initiative [4].

Where members of staff are less motivated, performance of their work goes down and usually the quality of services is compromised. Should it be an organization, then its viability remains questionable. It is therefore, important to find out what can be done to improve the overall interest of members of staff so as to promote staff welfare and improve performance. Studies have shown that when employees are motivated adequately, their performance increases and consequently production is as well positively increased [5,6]. General job satisfaction, general job happiness, satisfaction with salary and promotion, institution, educational background, and age of nurses’ youngest child were proved to be significant predictors of nurses’ intention to quit. In addition to this, Clinicians and clinic researchers can be guided by suggestions and insights from this study that organizational, motivation, and sociodemographic factors contribute to nurses’ intention to quit.

This study aims at exploring factors that influence motivation of staff at Adventist health facilities in Malawi to maximize their potential for work and improve their welfare. It is hoped that if members of staff are motivated to work and their welfare is taken care of then quality service delivery would be delivered to the patients.

## Methods

This was an operational research which utilized qualitative methods using group discussions and in-depth interviews. The researcher is a director for all the health facilities which are owned by the Seventh Day Adventist Church in Malawi and is based at the head quarters of the Church. He has a responsibility of ensuring that the health facilities are well managed and the general health status of the members within the church and beyond is well looked after. The Seventh Day Adventist (SDA) Church in Malawi offers various services in the country ranging from education, humanitarian services, publishing, health as well as spiritual nourishment as a primary goal.

### Study area

Malawi Union (MU) of SDA Church headquarters is based in Blantyre with a total of 32 staff members. Of these, 11 are females and the rest are males. The SDA church health systems operate in the country in accordance with the Malawi Government’s health facilities operating systems. The SDA health facilities complements what Malawi Government tries to achieve in matters relating to the nation’s health delivery services. However, as faith based institution, the health facilities are grouped together with the rest of the health facilities and they all belong to a non-Governmental body called Christian Health Association of Malawi (CHAM) which together offers 40% of the health care in Malawi. The Government and Non Governmental Organizations (NGOs) deliver health care in Malawi. Among the NGOs, faith based institutions ran health facilities but are subsidized by the Government. This study was performed in the following four main health facilities which are under Malawi Union of SDA Church; Malamulo hospital and Adventist Health Services (AHS) which has 17 clinics spread across the country. These two facilities have no membership to CHAM where as Adventist Health Centre – Lilongwe (AHC-L) and Blantyre Adventist Hospital (BAH) solely operate on private basis and do not get subsidy from Malawi Government.

These health facilities are scattered all over Malawi in different regions. These facilities have a total of 675 employees and of these, 553 are Seventh-day Adventists. Employment within the Adventist health facilities is open to all regardless of religion, gender, ethnicity or any background. Below is the brief description of the specific health facilities that were studied;

1. Malamulo mission hospital and Adventist Health Services serve rural population. Malamulo is located in the rural southern region of Malawi, 65 km south east of Blantyre City in Thyolo District. The hospital has 15 mobile sites with 2 health centres and collaborates with other NGOs. It is one of the teaching institutions in the country for allied health workers and has 300 beds. Malamulo hospital serves an estimated rural population of 70,000 whereas Adventist Health Services with 17 satellite clinics across Malawi has a catchment population of over 150,000. Malawi Government through the Christian Health Association of Malawi (CHAM) is funding both entities on the part of salaries for the staff but to a larger extent, Both facilities are self supporting through the clinics and revenues realized from patients’ fees is used for operations, purchasing medicines and equipment among others. Shortage of manpower, inadequate maintenance of facility structures and failure to recover operational costs from the respective service level agreement (SLA) with DHOs on time to run the activities are some of the challenges Malamulo and AHS are facing. However, with donor support clinic expansion has been commenced. The seasonal variations and shortage of manpower among the contributing factors affect the facilities’ patients’ uptake and income.
2. Blantyre Adventist Hospital (BAH) and Lilongwe Adventist Health Centre (AHC-L) are based in the commercial City of Blantyre and Lilongwe – Malawi’s capital city respectively. These facilities operate on private basis and were both initially established under the influence of Malamulo hospital basis in order to generate revenue base to help Malamulo and the rural satellite clinics cover the operating costs. This was because majority of patients accessing services at Malamulo and the rural clinics were not able to meet the cost of the services delivered to them. BAH has 36 bed capacity with urban population of 50,000 whereas AHC-L has at the moment 15 beds with a catchment population of 20,000. Both of these facilities continue to achieve progress in service delivery mission at very competitive levels with the rest of the reputable health facilities in the two cities.

### Sampling of informants

The informants were purposively selected based on the theoretical assumption that there were variations and range of understanding on different areas. The participants were selected based on their roles, experiences, years of service in the institution and with different age groups too. The selection of the participants was facilitated by the heads of the institutions and each participant was given a choice to either participate or not.

### Data collection

A total of 4 discussion groups were done with 29 participants grouped by gender, age and occupation. At least one member from each section of the health facility participated in the discussions. Of the 29 participants, 19 were males and 10 were females with the mean age of 38.5 years. Twenty were married and nine were singles. The participants’ number of children ranged from 1 to 7 with an average socio-economic status. The religious affiliations ranged from Christians, Moslems and none but majority were Adventists. The participants’ education ranged from primary to tertiary school levels. An interview guide was used during the discussions and covered topics such as interpersonal relations, workers’ satisfaction, internal controls, motivation and improvement of staff welfare (Table 1).

**Table 1:** Number of health facilities visited

**Discussion groups**	**Type of staff**	**Facility**	**Participants**	**Average age**
1.	H/workers & support staff	1	8	39
2.	H/workers & support staff	2	6	36
3.	H/workers & support staff	3	7	38
4.	H/workers & support staff	4	8	41

Note: Health workers comprised of nurses, clinicians, laboratory technicians and radiographers. Support staff comprised of cleaners, ward attendants/nursing assistants and ground workers.

The exercise was conducted from January – September 2011. Appointments to get permission to conduct the function were done through the heads of the facilities. Upon reaching the facility, the research team comprising of Malawi Union Health Director (the first author) and the Health Director for the region in which the facility is located commonly referred to as field or conference met the management team for the facility. The research team briefed the management the purpose of the visit and how to go through the procedure. The participants were told that a preliminary report would be given to the management team or head of their institution just before the researchers left their institution. The management team was told that final report would be given to them after the whole exercise and this too was agreed by both sides. All the selected and agreed participants and the researchers convened the meeting in the staff room. Participants were free to participate or not as their rights were respected. Here, the discussions were conducted in an open conversation. Three participants did not participate in the interviews due to their personal reasons.

The process was carried out in the following ways; the participants were gathered in the staff room. The researchers introduced the subject for discussion starting with the introductions, objectives, areas to be discussed and asked the participants to participate freely in the discussions. A time keeper, an observer and somebody to take the notes were also present. Thereafter a preliminary report of findings to the management team was given.

Participants anonymously agreed to start the discussions and end with prayers. The discussions were conducted in the morning as arranged by the heads of the facilities after discussing with the participants. Refreshments in the form of soft drinks and snacks were served at the expense of the field or conference responsible. The discussions lasted from 90 to 120 minutes after seeing that no more new information emerged.

### Data analysis

Data were analyzed according to the areas covered. The hand written notes which were later typed were managed manually. After thorough reading of all the notes, the content was analyzed thematically analyzed for identification of central issues by the two authors. Responses from the health facilities were grouped according to the areas that were being discussed. Data were compared to enrich the areas studied and be able to explain factors that motivate and demotivate members of staff to maximize their potential for work and improve their welfare.

### Methodological consideration

The first author’s prolonged engagement in the health facilities, experiences of the field visits and data collection activities were crucial for the trustworthiness of the study. To encourage openness among the participants we explained the issue of confidentiality and also asked the participants to agree not to share information from the discussion outside the group. Since the first author was a man as well as a representative of the hospital we made extra efforts to describe his role in the research process. During the analysis phase we actively made efforts to put our previous experiences within brackets to allow for unanticipated findings. In the research team we held regular peer debriefing and analysis sessions contributing with different competences, beneficial for interpreting the results.

We acknowledge the fact that for logistical reasons our study participants were sampled from the departments within the health facilities. This may be seen as limitation of our findings but we find no theoretical assumption supporting very different experiences in the more distant institutions.

### Ethical consideration

The Malawi National Health Sciences Research Committee in the Ministry of Health gave the permission to conduct the study. Further, consent was obtained from Malawi Union of SDA Church executive Committee coupled with permission from the heads of the institutions. In addition, respondents gave their oral informed consent and were all assured that information given would be treated with strict confidentiality. Based on the fact that participants were employees of the facilities under study and that the study did not extract them from their work stations, they opted to give verbal consent as opposed to written one.

## Results

There were several factors that influenced the motivation of the staff. Those factors were either motivating or demotivating. The motivating factors were spiritual nourishment of the church based institutions and long term benefits of members of staff and their families. The participants were quick to point out that job security, education and medical assistance and working in a Christian environment were all some of the motivating factors that made them to continue working in the church based health facilities. Pension benefits and spouse assistance during bereavement among others were some of the advantages of working in the church based health facilities. The demotivating factors that were mentioned by the participants were related to management. Participants felt that they were unfairly treated without respect. According to the participants, this meant poor and sometimes dangerous working conditions leading to dissatisfaction when the staff missed support in times of personal difficulties. Understaffing was observed as a challenge and was echoed by several members of staff from all the facilities. This resulted in making the existing staff work extra hours with the danger of providing poor quality services. It was said that quite a good number of people did apply for employment but when they joined the institution, they hardly stayed for a long time before they left for other areas. According to the members of staff, they felt that ‘the management does not have the staff welfare at heart’ as the root problems. Specific areas like working conditions, housing, allowances, training, communication, and personal support were highlighted. Another issue related to the loose of purpose, where Christian values were seen to be deteriorating and therefore undermining the mission of the institution.

### Motivating factors

#### Spiritual nourishment

Some members of staff expressed satisfaction by working in church based institutions in that their lifestyles were positively influenced. For instance, some participants stated that before joining the facilities, they used to drink and smoke heavily but following routine morning devotions and counsels of the facilities’ chaplains, they either reduced or completely stopped the practice. It was also mentioned that some had become more faithful to their partners after joining the health facilities.

### Long term assistance and benefits

The participants stated that the church work force enjoyed multiple benefits not only to themselves but also to their spouses, children and families in general. The benefits were education, pension, tax and bereavement assistance. For instance, the institutions assisted the members of staff to study further and their children up to the age of 24 and this enabled workers’ children to attain high level of education so long they worked hard regardless of the status in the organization. The workers themselves could as well be assisted to further their education up to the age of 57 so according to them, this was something that motivated them. At retirement age, members received a pension. If the worker died the spouse or children will continue to receive the pension. In addition, when a family member or the worker dies the institutions offer assistance according to the policy. Members working in the Adventist health facilities and other related church based institutions received at least 90% tax assistance. Participants stated that yes, the take home appeared small but increased their interest to work for the health facilities.

### Demotivating factors

#### Poor and sometimes dangerous working conditions

Water system and electricity were major issues in the health facilities. There was a serious problem of electricity though solar panels were installed and at one given time there was solar system of electricity. However, once the solar system got damaged, it was never repaired and the facility continued to experience problems. This became difficult when nurses conducted deliveries at night and this was coupled with shortage of gloves. Nurses could hardly conduct deliveries without gloves because doing so would put their lives at risk of contracting HIV infection among other communicable infections. These sentiments were also cited during the discussions.

Risk allowances were not being considered for some cadres such as those working in the laundry. Workers in the laundry section were constantly being exposed to risks of infections since they were manually doing their work and it was said to be high time for the hospital management to pay some attention to this or perhaps buy a laundry machine to safe guard staff welfare.

Housing was another issue that came up. It was said that inadequate and poor maintenance of housing posed a challenge to the existing staff and those willing to join the institutions. Much as the institutions wished to employ more workers but the houses were inadequate for the new staff, such that prospective staff failed to take up their appointments at the health facilities.

### Dissatisfaction with management of allowances and contracts

It was observed among staff that allowances were reduced, revised several times or not paid for on time and this de-motivated the members of staff. The allowances stressed in the discussions that were reduced included education assistance, call allowances and allowances accrued during holidays (locums). Staff members were meant to understand that reduction of the allowances was facilitated by the Malawi Union (the headquarters of the Seventh Day Adventist Church in Malawi) an understanding which needed clarification bearing in mind these health facilities particularly Malamulo hospital was undergoing economic difficulties at the time. Staff on contract for years posed a threat in that members of staff felt unsecured. Initially, it was mentioned that when the hospital was underwent financial difficulties, some employees were retrenched and others were put on contract. The understanding was that when the hospital’s financial situation improved, employees on contract would be put on permanent employment status, a development which has not been done even when the hospital finances had improved. Staff on contracts was not getting the benefits like others who were on permanent employment. It was mentioned that some members of staff were demoted without proper reasons and if the demotions were done, the said staff were maintained in the same department under someone who got promoted and was once a junior staff. This behaviour was frustrating and unwelcome according to the members of staff.

It was reported that there were differences between old and new staff in that the treatment given to the new staff was different from the treatment that was given to the old staff. For instance, the new staff members were said to have been recruited with full benefits such as maternity leave grants, house rents and education assistance among others whereas the old staff had to undergo probation period and it was soon after completing their probation period when they had to receive full benefits. It was also mentioned that retirement benefits kept on changing without clear directions.

### Problematic communication practices

Interpersonal relationship was viewed as a challenge. When asked to elaborate on this, staff members expressed that many times people worked under fears in that if they did not comply with what they were told to do, they would be fired. They cited an example where some members of staff were reprimanded in public or in presence of patients for not doing certain things well. They felt intimidated by this especially when this behaviour was being demonstrated by the senior staff of the hospital. The sour relationship was triggered by the fact that members of staff received unequal treatment depending on their levels or positions at work. Those with low positions were unfairly treated compared to those with high positions. Some doctors were more favored than others. The same applied to the fact that young and beautiful nurses/female employees were more favored than the older ones – a development that was said to be worrisome at some of the health facilities.

Communication with staff members regarding operations of the hospital was observed as a challenge. It was mentioned that staff meetings were no longer done regularly; as such members of staff had difficulties in airing out their views. These members of staff were asked to execute things without being told in advance and felt the decisions were mostly made by one person. The management was able to take certain decisions without making consultations with the rest of the staff members. This included changes that took place within the institution; “*one only sees things happen without being communicated*”. This resulted also in decisions which were perceived unfair. Staff members expressed for example concerns that they felt that the recruitment of some new staff was based on relations and the policy was not followed.

### Lack of opportunities for continuing professional development

It was stated that members of staff hardly accessed seminars and formal training. The shortage of staff was stated to have contributed to their situation of not being considered for further training. The continuing professional development (CPD) which is a requirement for any health professional in Malawi was a worrisome issue to the nurses at the facility. Participants felt that they were not considered in this aspect. Members of staff would wish to visit sister institutions to learn on different modalities of doing things, but the opportunity to do so was not freely granted and if granted it was said to be given to the same people over and over again – a happening which is demoralizing according to the staff members.

Staff were also not given time to go for further studies and if one insisted he would be advised to resign. However, senior members of the management were said to be allowed to go for studies without problems.

### Missing support in times of personal difficulties

It was mentioned that members of staff were sidelined especially when it came to what was important for them. They cited the fact that they felt that they were not being supported by those in powers such as the matron, medical director and a few other heads of department. For instance; it was difficult to access loans, at times deductions were made from those on sick leave without prior communication.

The facility’s social welfare, an employee grouping which seeks to ease social problems such as bereavements among others experienced by members of staff of all walks of life in that organization appears to be discriminatory. Individuals felt that the association benefited a selected few belonging to high ranks with high qualifications whereas those with lower qualifications and the support staff were least considered when they had problems. There were stories where staff in difficult personal situations had not been supported by the institution but rather reprimanded for not reporting for duties. When loans sometimes were given it was felt the monthly deductions were done without considering what he can remain with to feed his family. Loans were given to members of staff without considering the period of service of an employee and the capacity to repay it. Staff members were of the opinion that the institution would have to shoulder the expenses in the event that the employee dies before accumulating adequate benefits to offset the loans.

### The institution on the way to lose its purpose

According to that participants, this meant that some facilities lost their vision and so they could not realize their goals. Much as the facility was responsible for offering medical care to the communities around, it was important to understand that primarily the institution was responsible for evangelism, in other words ‘God first’. This notion received an enormous support from almost all participants in the meeting. Several examples were given to exemplify this such as not having evangelistic outreach, no efforts to help the destitute or visit the sick as was the case in the past.

As a result the spiritual aspect of the staff members was said not to be healthy. It was mentioned that attendance to the worship has gone down and people related it with actions of the management and their attitude. People were unhappy and felt as if they were working in a secular institution. The spiritual health was affected negatively when members of staff with dubious behaviors took turns in sharing the word of God instead of being role models. This situation affected not only the Adventist Christians but also members of other denominations. Therefore, instead of witnessing for Christ, the facility was not doing what it was supposed to do. It was further observed that on Sabbath the operations of the hospital went on as week days- a development which did not go well according to the doctrines of SDA church.

Morning devotion was a challenge. It was reported that there was no morning devotions at some of the facilities since members of staff report late for duties and do not observe the working conditions of the facility. Here, even one sister in-charge acknowledged that she usually reported late for duties as well because most of the times she was all by herself and usually overworked. In addition, evangelism meetings were not done in the year this study was being conducted and appeared not to be put in the annual plans. As a consequence of no morning devotion taking place it was hard to think of preaching the gospel to other people.

## Discussion

The paper highlights issues pertaining Adventist health facilities in Malawi. Implications are similarly presented as per discussions in the order of the following prevailing events.

The study has showed that much as the members of staff expressed the concerns regarding their working conditions, they were quick to point out that job security, education and medical assistance and working in a Christian environment were some of the motivating factors that made them to continue working in the church based health facilities. This is consistent with what was established before [3] where social factors were perceived to impact positively on people’s desire to work and increased production.

Pension benefits and spouse assistance during bereavement among others were some of the advantages of working in the church based health facilities. The finding may imply that employees were working towards self actualization as individuals; at family levels, children would have long term benefits in terms of education and sickness; employees and their spouses would also benefit during their old age even if one of them dies, who ever remains would still be supported by the pension benefits. Further, from the findings, one may conclude that salary alone may not be the key determinant of motivation.

The study revealed that shortage of manpower was a cross cutting issue among all the health facilities though with varied degrees. This may have several implications; performance of the facility may be reduced leading to poor quality services offered to patients; health workers who remain committed to their work particularly nurses may be overworking which may result into burn outs; over working may influence the health workers’ attitude negatively ending into seeing patients as nuisance preventing them from resting and may be shouting at patients unnecessarily. At times, there may be possibility of preventable accidents such as injuries and inadequate adherence to infection prevention practices among others. As a result of this health workers may contract infections such as HIV or hepatitis and other communicable diseases. This finding is congruent with what was found before [7,8]. In these studies, it was found that health workers with long working hours were prone to developing burn out syndrome. This consequently affected their health and performances in many aspects.

The importance of adequate safe water and acceptable accommodation can not be underestimated. These findings may suggest that the health facilities are struggling financially, poor planning and prioritizing on the part of the management, workers’ working conditions may have shortfalls to be amended, these facilities may not attract new staff and let alone patients and principles of infection prevention practices may be compromised. The effects of these may have long term effects on both patients and health workers [9,10]. Many qualified health workers would like to have decent accommodation with acceptable basic amenities such as water and electricity among others. Consequently, facilities without these amenities would facilitate exodus of health workers to places with favorable working conditions described as having good and green pastures.

Staff welfare and motivation were perceived not as important by some of the administrators of the health facilities. Treatment to members of staff by the management was different depending on the cadre or position and this did not go well with some of the staff members, the study has established. However, much as staff members may be motivated to continue to work hard due to many factors, the nature of their profession may be one of the driving forces for them to fight on. Further, the organization of the facility has a great role to play as far as staff motivation and welfare are concerned [11-13]. Additionally, seeing each other as partners in development has shown in itself as an important motivating factor (reference). Respect among each other, realizing one’s potential and appreciations of what others are doing are of great significance in development.

It was established through this study that spiritual nourishment of the health facilities affected the workers positively. The finding is congruent with what was found before [14]. In that study, it was observed that spitual care facilitate patients care, even if the patients do not recover fully from their illness but can easily accept their situation and if need be they are able to accept their own death. This may have several implications; individuals’ life styles may have changed in that people who used to drink or smoke could reduce or stop completely, family welfare and economy may be positively influenced as money meant for food may be directed at that and individuals may stay long with their families. Further, patients or clients in general accessing services in the facilities may be well attended to.

The study showed poor interpersonal relationships among various levels of staff members and let alone communication. Staff members were not free as they discharged their duties for fear of being reprimanded and consequently the management did not communicate issues to health workers on time. This may suggest that in certain situations, there were no transparency and accountability among staff members and their management; patients’ care may be affected; work may have been seen as a means to an end and not a means in itself. In other words, health workers might be experiencing problems which may affect their health because they are not able to share them with others for solutions.

The study has established the spiritual decline of the institutions though in abstract form but expressed by the health workers. This has been indicated by the fact that the morning devotions were not regularly done in some of the health facilities and in some they were not done at all. This may have several implications; spiritual weakness of the institutions’ leaders; some workers may not be Christians at all; shortage of staff coupled with increased workload with no or limited time to conduct devotions. As a result of this, there might be no time for praying with patients and patients care may be affected. Studies before have established that spiritual care is of utmost importance in as far as patient care is concerned [14,15]. More also being Adventist institutions, spiritual nourishment is enshrined in the policy of the church system and forms an integral part of the church’s doctrines and its existence.

Financial constraints and failure to capture data from some of the health facilities in very remote areas of the country were the limitations. However, the study has the strength of exploring the motivating factors among members of staff working in Adventist health facilities in Malawi to maximize their potential for work and improve their welfare.

## Conclusions

The study has established that working in Adventist health facilities has multiple benefits at individual and family levels. Education and medical assistance including pension benefits and spiritual nourishments have been some of the advantages of working in the church system. In addition, unfair treatment, poor accommodation, poor communication and interpersonal relationships were pinpointed as challenges experienced by the members of staff. Based on these findings, the following recommendations were made; issues seemed as advantageous to staff members should be promoted in the church system operating policies. However, conditions of services for the Adventist health facilities need to be revised and implemented accordingly; training of staff for further facility development to be intensified, exchange visits of staff from one institution to the other to foster sharing of knowledge should be offered periodically and dialogue/communication between management and staff needs to be strengthened. Interaction among health facility staff and churches and their respective pastors including the communities surrounding the facilities should be strengthened to enhance spiritual nourishment and growth. A further study is needed to look at the community perceptions towards the services offered in the Adventist health facilities.

## Abbreviations

AHC-L: Adventist Health Centre – Lilongwe
AHS: Adventist Health Services
BAH: Blantyre Adventist hospital
CHAM: Christian Health Association of Malawi
CPD: Continuing Professional Development
DHO: District Health Office
ESCOM: Electricity Supply Commission of Malawi
ICU: Intensive Care Unit
MU: Malawi Union
NGO: Non Governmental Examinations
SDA: Seventh Day Adventist
SLA: Service Level Agreement
